# Social network characteristics, self-management behavior, and the mediating role of self-efficacy among diabetes patients: a multicenter cross-sectional study

**DOI:** 10.3389/fendo.2026.1934060

**Published:** 2026-09-24

**Authors:** Xixuan Cai, JianJiang Pan, Gang Li, Tao Chen, Zhou Fang, Zhangqing Ren, LongMeng Peng, JingJing Huang, XiuHua Gong, QingXia Chen, BaoJi Wang, LuSha Li, LiYing Chen

**Affiliations:** 1 Department of General Practice, Sir Run Run Shaw Hospital, School of Medicine, Zhejiang University, Hangzhou, Zhejiang, China; 2 Department of General Practice, Shaoxing People’s Hospital, Shaoxing, Zhejiang, China; 3 Jianqiao Community Health Service Center, Hangzhou, Zhejiang, China; 4 Shaoxing Second Hospital Lizhu Branch, Shaoxing, Zhejiang, China; 5 Department of General Medicine, Chengdong Community Health Service Center, Chengdu, Sichuan, China; 6 Community Health Service Center of Xiasha Street, Hangzhou, Zhejiang, China; 7 Yangliuxue Town Health Center, Binzhou, Shandong, China; 8 Shibali Town Health Center, Bozhou, Anhui, China; 9 Xinhe Community Health Service Center, Xi’an, Shaanxi, China

**Keywords:** Chinese population, diabetes mellitus, type 2, self-efficacy, self-management behavior, social networks

## Abstract

**Backgroud:**

Social isolation (structural social network deficit) is closely linked to poor health outcomes in patients with diabetes. Existing studies often focus on broad isolation or single-dimensional factors, lacking targeted exploration in the Chinese population.

**Objectives:**

To explore the associations between social network characteristics and self-management behavior in patients with diabetes, and assess the mediating role of self-efficacy in this association.

**Methods:**

A multi-center cross-sectional study was used to recruit 2, 358 patients with diabetes. The assessments included social network characteristics, self-efficacy, and self-management behavior. Pearson’s correlation analysis was employed to examine the relationships between these variables. Regression models were utilized to assess the mediating effect of self-efficacy, with the bootstrap method applied to confirm the significance of the mediation pathways.

**Results:**

Among the 2, 358 participants, the average diabetes management score was 36.51. Regression models indicated that both family network size (*β* = 0.251, 95% CI: 0.048–0.455, *p* = 0.016) and friend network size (*β* = 0.325, 95% CI: 0.147–0.504, *p* < 0.001) were significantly associated with better self-management behavior in patients with diabetes. Family support (β = 0.558, 95% CI: 0.406–0.710, *p* < 0.001) and friend support (β = 0.624, 95% CI: 0.484–0.764, *p* < 0.001) also showed strong positive effects on self-management behavior in patients with diabetes. Pearson’s correlation analysis revealed significant positive associations between social network characteristics, self-efficacy, and self-management behaviors in patients with diabetes (*p* < 0.001). The mediating role of self-efficacy was confirmed, with significant indirect effects observed for both family and friend support.

**Conclusions:**

This study highlights the significant influence of social network characteristics, particularly social support, on self-management behavior in diabetic patients. Family and friend support positively impact self-management behaviors, and self-efficacy mediates these relationships.

## 1 Introduction

Type 2 diabetes mellitus (T2DM) is a chronic and progressive metabolic disorder that poses a substantial burden on global healthcare systems (1). Effective self-management is critical for preventing complications and improving health outcomes in individuals with T2DM, requiring patients’ active participation in treatment regimens, lifestyle modifications, and regular monitoring of key health parameters such as blood glucose (2, 3). While medical treatments, lifestyle interventions, and educational programs have been the focus of diabetes care, there is growing recognition of the critical role social networks play in supporting diabetes management (4).

Social networks—encompassing family, friends, healthcare providers, and community groups—are increasingly regarded as fundamental components of chronic disease self-management (5). Social support, both from healthcare professionals and peer groups, has been well-documented to positively impact diabetes care, though the influence of informal social networks (e.g., interpersonal relationships in one’s immediate circle) remains less thoroughly explored. Early studies, including those by van Dam et al., suggested a beneficial association between social support and glycemic control, but methodological limitations hindered firm conclusions (6). Recent research has aimed to address these gaps by identifying key mechanisms through which social networks influence diabetes management, such as knowledge sharing, resource access, and interpersonal navigation—all essential for effective self-management (5). Furthermore, larger, more supportive social networks, particularly those providing informational and emotional support, have been linked to improved diabetes outcomes, including better self-management behaviors (5). for instance, Koetsenruijter et al. found that emotional and informational support networks correlated positively with enhanced self-management capabilities, especially in low-income populations (7). Beyond managing existing T2DM, emerging evidence indicates social networks may also play a role in prevention, as T2DM risk factors like obesity and shared health behaviors (e.g., diet, physical activity) can spread through social networks (8, 9).

Despite these insights, social networks—while recognized as key components of the social environment that facilitate diabetes self-management—remain understudied in critical areas, leaving obvious research gaps. Specifically, the mechanisms linking social network characteristics to diabetes self-management and the multidimensional assessment of social networks are insufficiently understood. Reviews have emphasized the need for theory-based, mechanism-focused research to clarify how social network structure and function influence self-management (5, 10, 11). A meta synthesis on social networks and chronic illness identified broad mechanisms (e.g., network navigation, negotiation, collective efficacy) but noted limited specification of these mechanisms for diabetes self-management behaviors (5, 10). A narrative review in Diabetologia further highlighted that current network measures are heterogeneous and overly simplistic, calling for more detailed, harmonized assessments of social network structural and functional characteristics to inform targeted interventions (10).

These research gaps are even more prominent in Chinese populations. Most existing Chinese studies on diabetes and social networks focus on single-dimensional indicators (e.g., social isolation index, family support) rather than comprehensive assessments of social network structure and function. For example, a CHARLS-based analysis on the association between social isolation and HbA1c operationalizes isolation via a composite “social isolation index” without detailed network mapping (12). Studies on family support in rural China have focused primarily on family function and family self-efficacy, neglecting broader network topology and non-family ties (13). While some randomized trials have found that social network-based health education improves diabetes self-care behaviors (e.g., diet, blood glucose testing), they have not systematically analyzed social network structure or underlying mechanisms (14). Additionally, rural China research has shown that family support mediates the effects of family function and self-efficacy on self-management, but this finding is confined to intra-family dynamics and cannot be extended to broader social network contexts (13). A scoping review of peer support and social network interventions in sub-Saharan Africa similarly concluded that mechanisms linking network characteristics to outcomes remain unclear and understudied (11), echoing the universality of this gap.

Self-efficacy, defined as the belief in one’s ability to successfully perform behaviors necessary to achieve desired health outcomes, plays a central role in promoting health behaviors. Enhancing self-efficacy has been shown to improve health behaviors in individuals with chronic conditions such as hypertension, chronic obstructive pulmonary disease (COPD), and cardiovascular diseases (15). However, the role of social networks in shaping self-efficacy and how this influences diabetes self-management behaviors requires further exploration.

This study seeks to address these gaps by investigating the relationship between social network characteristics and self-management behaviors in individuals with T2DM, with a focus on the mediating role of self-efficacy. Using data from the PDHMP-China study, a large population-based cohort of individuals with diabetes, we aim to examine how different dimensions of social support—such as the size, density, and type of social network—are associated with self-management behaviors. Additionally, we will explore whether self-efficacy mediates these relationships. By shedding light on these dynamics, this research aims to contribute to the development of more effective interventions that leverage social networks to enhance diabetes care and improve health outcomes across diverse populations.

## 2 Methods

### 2.1 Study population

This population-based cross-sectional study utilized baseline survey data from the Project of Diabetic Health Management and Promotion in China (PDHMP-China). PDHMP-China is a nationwide initiative dedicated to comprehensively evaluating and optimizing diabetes management strategies through interdisciplinary research, focusing on lifestyle modification, disease self-management education, and the development of social support systems.

The PDHMP-China project employed a multi-stage stratified cluster sampling design to ensure representativeness and feasibility across diverse primary healthcare settings.First stage: Given China’s vast territory and uneven socioeconomic development, the mainland was stratified into four regions (East, Central, Northeast, and West) based on national development and regional coordination standards. A total of 53 potential community health service centers (CHSCs) were contacted, and 26 centers from 12 provinces were successfully enrolled: East: Zhejiang, Guangdong, Jiangsu, Shandong;Central: Henan, Hunan, Anhui;Northeast: Liaoning;West: Yunnan, Shaanxi, Xinjiang, Qinghai;Second stage: Within each selected CHSC, all registered patients with diabetes were identified from local household registration and electronic medical records (EMRs). A sampling frame was created by assigning a unique identifier to each eligible patient, and a random number generator was used to select participants. To ensure adequate sample size and representativeness, each CHSC was required to recruit a minimum of 150 patients with diabetes.

Inclusion criteria were:

- aged 18–80 years
- a confirmed diagnosis of type 2 diabetes mellitus (T2DM), defined as either a self-reported physician diagnosis, a fasting plasma glucose (FPG) level ≥ 7.0 mmol/L, or a glycated hemoglobin (HbA1c) level ≥ 6.5% during routine follow-up (11)
- provision of written informed consent and voluntary participation.

Exclusion criteria were:

- special types of diabetes (e.g., type 1 diabetes, gestational diabetes);
- severe diabetic complications (e.g., advanced diabetic nephropathy, proliferative retinopathy)
- other severe comorbidities (e.g., decompensated heart failure, end-stage renal disease, respiratory failure)
- recent cancer treatment (chemotherapy or radiotherapy within the past 6 months);
- language or cognitive barriers preventing independent or assisted questionnaire completion.

### 2.2 Data collection and management across multiple sites

Data were collected between November 2023 and March 2024 at all 26 participating CHSCs. A standardized, centrally developed data collection protocol was uniformly implemented across all sites to ensure data quality and consistency:

#### 2.2.1 Sample size calculation

The sample size was calculated based on the expected prevalence of social network isolation among patients with diabetes, which was estimated to be 22.67% according to previous studies (16). The formula for sample size estimation in a cross-sectional study with a complex sampling design was used: n = (Zα/2² × p × (1−p)/δ²) × deff, where Zα/2 was 1.96 corresponding to a 95% confidence interval, p was the expected prevalence of social network isolation (0.2267), δ was set at 10% of the expected prevalence (0.02267) as the allowable error, and deff was 1.5 as the design effect for complex sampling. The calculated minimum sample size was approximately 1966 individuals. Considering a 10% non-response rate due to incomplete questionnaires or missing key variables, the required target sample size was adjusted to 2184 participants.

#### 2.2.2 Participant recruitment and enrollment

At each CHSC, trained research coordinators identified eligible patients from the center’s diabetes registry. Eligible patients were approached during routine outpatient visits or community health screenings. Study information was provided in plain language, and written informed consent was obtained prior to any data collection activities.

#### 2.2.3 Data collection procedures

Sociodemographic characteristics, medical history, social network properties (structural and functional dimensions), self-efficacy, and self-management behavior were collected via face-to-face interviews using a structured electronic questionnaire. Clinical measurements (FPG, HbA1c, body mass index, blood pressure, etc.) were performed by certified nurses or laboratory technicians following standardized operating procedures (SOPs).

#### 2.2.4 Staff training and quality control

All research staff (interviewers, nurses, data managers) completed a 2-day centralized training program on study protocols, questionnaire administration, and data entry. On-site supervision and regular data audits were conducted by the central project team to monitor adherence to SOPs and identify missing or inconsistent data.

#### 2.2.5 Data management and security

Collected data were entered in real time into a secure, password-protected electronic database at each site. A central data management team at the project headquarters performed weekly data checks, including range checks, logical consistency checks, and missing value assessments. Discrepancies were resolved by cross-referencing with original records and contacting local research staff for clarification. All data were stored on encrypted servers with restricted access to ensure confidentiality.

A total of 4065 patients with diabetes were initially recruited. After excluding incomplete questionnaires (missing >20% of core variables) and invalid responses (e.g., inconsistent answers to logical check items), the effective response rate was 82.65%, resulting in 3361 valid observations. For the current analysis, an additional 1003 participants were excluded due to missing data on key variables (social network characteristics, self-efficacy, or self-management behavior), leaving a final analytic sample of 2358 participants.

### 2.2 Social network questionnaire

Trained interviewers conducted a systematic face-to-face interview to assess the egocentric social network properties of participants. All social network properties were based on the social network status of the year before baseline evaluation.

#### 2.2.1 Structural characteristics of the social network

Our primary measure of social networks was the abbreviated Lubben Social Network Scale (LSNS-6) (17). This scale includes six questions: three key questions assess the size of social networks attributed to family ties, and a parallel set evaluates friendship ties. The LSNS-6 measures the size of the respondent’s active social network (i.e., relatives or friends seen or heard from at least once a month), perceived support network (i.e., relatives or friends who could be called on for help), and perceived confidant network (i.e., relatives or friends with whom the respondent could discuss private matters). Each LSNS-6 question is scored on a scale from 0 to 5. The total score is the equally weighted sum of these six questions, with scores ranging from 0 to 30. We also summed the subscale scores for family and friends, with results ranging from 0 to 15. Higher scores indicate larger social networks. The validated LSNS-6 has been widely used and has established a cut-point of 12 for optimal overall sensitivity (18).

#### 2.2.2 Functional characteristics of the social network (social network support)

Participants were asked about the support they received from family and friends in managing their diabetes, using a 5-point Likert scale, where 1 = “never” to 5 = “frequently”.They were constructed on a model of four arenas of social support: 1) Instrumental support (tangible goods and actions), 2) Informational support (knowledge), 3) Emotional support and 4) Appraisal support (feedback regarding accuracy of beliefs and appropriateness of actions) (19). Higher scores indicate greater support from family and friends. More detailed information on the types of support assessed in this study is provided in **Supplementary Table S1** in the Data Supplement. These questions were developed and tested based on prior studies of patients with diabetes and family supporters (20–22).

### 2.3 Assessment of diabetes self-care activities

The Summary of Diabetes Self-Care Activities (SDSCA) questionnaire, developed by Toobert and Glasgow, offers researchers a straightforward tool for evaluating diabetes self-care activities over the past 7 days or the past month (23). The questionnaire focuses on various aspects of self-care, including diet, exercise, blood sugar testing, foot care, and medication monitoring, by assessing the absolute frequency or consistency of diabetes-related activities. Validation studies of the SDSCA have been conducted in the U.S. and Korea (24, 25). Participants were asked to report the number of days in the past week they engaged in each self-care activity using an 8-point Likert scale (0–7), where 0 indicates “never done” and 7 indicates “every day.” Higher scores signify better compliance with diabetes self-care activities, while lower scores reflect poor self-care performance. Behavior levels were classified based on the indicator value, with the indicator value (%) calculated as actual score/theoretical maximum score × 100%. An indicator value ≤ 40% represents poor behavior, 40%–80% indicates moderate behavior, and > 80% represents good behavior.

### 2.4 Assessment of diabetes self-efficacy

Participant self-efficacy was assessed using the Stanford Self-Efficacy for Diabetes Scale. This scale consists of eight items that measure a participant’s confidence in performing specific activities, including eating meals every 4–5 hours, adhering to a diet, selecting appropriate foods, exercising, preventing blood glucose fluctuations, determining when to consult a doctor, and managing diabetes to avoid interference with daily life. Scores on the Self-Efficacy Scale range from 1 to 10, with 1 indicating “not at all confident” and 10 indicating “totally confident.” Higher scores represent greater self-efficacy. The total score is calculated as the mean of the individual item scores. The scale has demonstrated good internal consistency reliability, with a Cronbach’s alpha greater than 0.8 (26).

### 2.5 General measurements

Participants’ demographic characteristics, including gender, age, marital status, educational level, current residence, Ethnic, and occupational status, socioeconomic status, living arrangements were acquired through face-to-face interviews. low education (no education, primary education, and lower vocational education). intermediate education (intermediate vocational education, higher secondary education, and vocational education). high education (higher professional education, university).

### 2.6 Statistical analysis

Prior to formal analysis, the normality of continuous variables was examined using the Kolmogorov-Smirnov test. Variables including social network characteristics, self-efficacy, and self-management behavior scores were approximately normally distributed; therefore, parametric statistical methods were used for subsequent analyses. Firstly, this study conducted a statistical description of sociodemographic characteristics and key variables. Categorical variables were summarized with frequencies (percentages). Continuous variables with normal distribution were presented as means (standard deviation, SD), which was consistent with their distribution characteristics. Secondly, Pearson’s correlation analysis was employed to assess bivariate correlations among social network properties, self-efficacy, and self-management behavior, because all these variables were continuous and normally distributed.

Linear regression models were used to explore the associations between these continuous variables, as the outcome variable (self-management behavior) was continuous and normally distributed, satisfying the assumptions of linear regression. Mediation analyses were conducted using the mediation package in R software, developed by Imai et al. (27), which is widely used in medical (28) and epidemiological research (29). The mediation package facilitated linear regression models, where potential mediators served as dependent variables, with other covariates as independents. Linear regression models also examined self-management behavior as the outcome variable, along with mediators and covariates as independents. The use of linear regression in mediation analysis was justified by the continuous and normally distributed nature of the mediator and outcome variables. Direct and indirect effects, with 95% confidence intervals, were estimated using quasi-Bayesian Monte Carlo methods with 5000 simulations each (30).

To verify the robustness of the primary analysis results and further explore the association between social network characteristics and self-management behaviors in different subgroups, several sensitivity analyses were conducted. First, considering the potential influence of diabetes duration, medication use, and complications on self-management behaviors, we further adjusted the fully adjusted models to account for these factors. Secondly, analyses were performed based on the individual domain scores of self-management behaviors, including diet, exercise, blood glucose monitoring, foot care, and adherence to self-care recommendations. Finally, subgroup analyses were conducted according to gender and age.

All statistical tests were two-sided, with significance set at p < 0.05. All analyses were conducted using SAS software V.9.4 for Windows (SAS Institute, Cary, NC, USA) and R software V.3.5.2 (R Core Team, 2018).

## 3 Result

### 3.1 Participant recruitment and characteristics

#### 3.1.1 Sociodemographic, lifestyle and clinical characteristics of the participants

A total of 2, 358 diabetic patients were enrolled, including 1, 140 females and 1, 218 males, with a mean age of 62.4 years. The majority were married (91%) and of Han ethnicity (95%). Most participants had a low education level (68%) and were either retired (37%) or not employed in paid work (29%). Most participants resided in the eastern region of China. Economically, 19% were classified as very poor, and 16% as poor. The majority lived with family or others (92%). In terms of lifestyle, 19% were current smokers, and 27% were current drinkers. Over half of the participants (53%) had hypertension. Detailed participant characteristics are summarized in **Table 1**.

**Table 1:** Sociodemographic characteristics variables of participants.

Characteristic	Overall,	Poor behavior,	Moderate and good behavior	p
	N = 2, 358	N = 805	N = 1, 553	
Age (years)	62.4 ± 11.6	62.5 ± 11.2	62.4 ± 11.8	0.776
Sex				0.169
female	1, 140 (48%)	405 (50%)	735 (47%)	
male	1, 218 (52%)	400 (50%)	818 (53%)	
Smoking Status, n(%)				0.032
current	437 (19%)	171 (21%)	266 (17%)	
former	133 (6%)	49 (6%)	84 (5%)	
never	1, 788 (76%)	585 (73%)	1, 203 (77%)	
Alcohol Consumption, n (%)				0.029
never	1, 729 (73%)	568 (71%)	1, 161 (75%)	
current	629 (27%)	237 (29%)	392 (25%)	
Marital Status, n (%)				0.100
Married	2, 140 (91%)	725 (90%)	1, 415 (91%)	
Single	57 (2%)	27 (3%)	30 (2%)	
Widowed	161 (7%)	53 (7%)	108 (7%)	
Ethnicity n (%)				0.948
Han	2, 234 (95%)	763 (95%)	1, 471 (95%)	
Non-Han	124 (5%)	42 (5%)	82 (5%)	
Educational Level, n (%)				0.019
high education	287 (12%)	82 (10%)	205 (13%)	
intermediate education	469 (20%)	147 (18%)	322 (21%)	
low education	1, 602 (68%)	576 (72%)	1, 026 (66%)	
Employment status, n (%)				<0.001
employed	481 (20%)	166 (21%)	315 (20%)	
no paid job	690 (29%)	291 (36%)	399 (26%)	
not known	35 (1%)	14 (2%)	21 (1%)	
retired	880 (37%)	241 (30%)	639 (41%)	
unemployed	272 (12%)	93 (12%)	179 (12%)	
Household registration, n (%)				<0.001
northeast	109 (5%)	36 (4%)	73 (5%)	
eastern	1, 130 (48%)	395 (49%)	735 (47%)	
westward	292 (12%)	142 (18%)	150 (10%)	
Central	827 (35%)	232 (29%)	595 (38%)	
Income, n (%)				<0.001
Very poor	458 (19%)	212 (26%)	246 (16%)	
Poor	378 (16%)	95 (12%)	283 (18%)	
Fair	459 (19%)	165 (20%)	294 (19%)	
Good	548 (23%)	161 (20%)	387 (25%)	
Very good	515 (22%)	172 (21%)	343 (22%)	
Living arrangement, n (%)				0.274
living alone	188 (8%)	71 (9%)	117 (8%)	
with family or others	2, 170 (92%)	734 (91%)	1, 436 (92%)	
Hypertension, %	1, 252 (53%)	439 (55%)	813 (52%)	0.314
Diabetes duration				<0.001
Less than 5 years	1, 078 (46%)	430 (53%)	648 (42%)	
5–10 years	640 (27%)	204 (25%)	436 (28%)	
More than 10 years	640 (27%)	171 (21%)	469 (30%)	
Treatment				<0.001
Oral Medication	1, 674 (71%)	576 (72%)	1, 098 (71%)	
Insulin-Related	579 (25%)	165 (20%)	414 (27%)	
Non-medication	105 (4%)	64 (8%)	41 (3%)	
Complications of diabetes, %	595 (25%)	177 (22%)	418 (27%)	0.009

#### 3.1.2 Diabetes management, self-efficacy and social support status of participants

The average diabetes management score was 37, and the self-efficacy score was 58. The average Social Network Score was 12.4, with a Family Network Score of 6.7 and a Friend Network Score of 5.7. The Family Social Support Score was 14.00 ± 3.56, and the Friend Social Support Score was 9.61 ± 4.03. Detailed participant characteristics are summarized in **Table 2**. **Figure 1** illustrates the mean and standard deviation of various support types from family and friends, including emotional, informational, instrumental, and appraisal support. It highlights that family network support has the highest mean score. **Figure 2** compares the mean scores of these support types between poor and moderate to good behaviors, revealing the differences in support perceived across these two categories.

**Table 2:** Critical characteristics variables of participants.

Characteristic	Overall,	Poor behavior,	Moderate and good behavior	*p*
	N = 2, 358	N = 805	N = 1, 553	
Diabetes Management Score	36.51 ± 13.55	22.08 ± 6.43	43.98 ± 9.66	<0.001
Diet score	16.3 ± 5.51	11.72 ± 4.70	18.7 ± 4.23	<0.001
Exercise score	5.84 ± 4.06	3.33± 3.15	7.15 ± 3.86	<0.001
Blood glucose testing score	3.89 ± 4.12	1.37 ± 1.84	5.20 ± 4.36	<0.001
Foot care core	4.81 ± 4.98	1.34 ± 2.24	6.61 ± 5.05	<0.001
Medication Use Score	5.61 ± 2.46	4.33 ± 3.16	6.28 ± 1.64	<0.001
Functional characteristics of the social network				
Social network Support Score	23.60 ± 6.25	21.79 ± 6.11	24.54 ± 6.12	<0.001
Family network Support,	14.00 ± 3.56	13.32 ± 3.73	14.35 ± 3.42	<0.001
Friend network Support,	9.61 ± 4.03	8.47 ± 3.80	10.19 ± 4.02	<0.001
Structural characteristics of the social network				<0.001
Social Network Score (total)	12.46 ± 5.61	11.71 ± 5.90	12.85 ± 5.41	<0.001
Family Network Score	6.75 ± 2.99	6.47 ± 3.11	6.89 ± 2.92	0.001
Friend Network Score	5.71 ± 3.49	5.24 ± 3.67	5.95 ± 3.36	<0.001
Self efficacy	58.25 ± 14.40	52.08 ± 16.06	61.45 ± 12.30	<0.001

**Figure 1:**
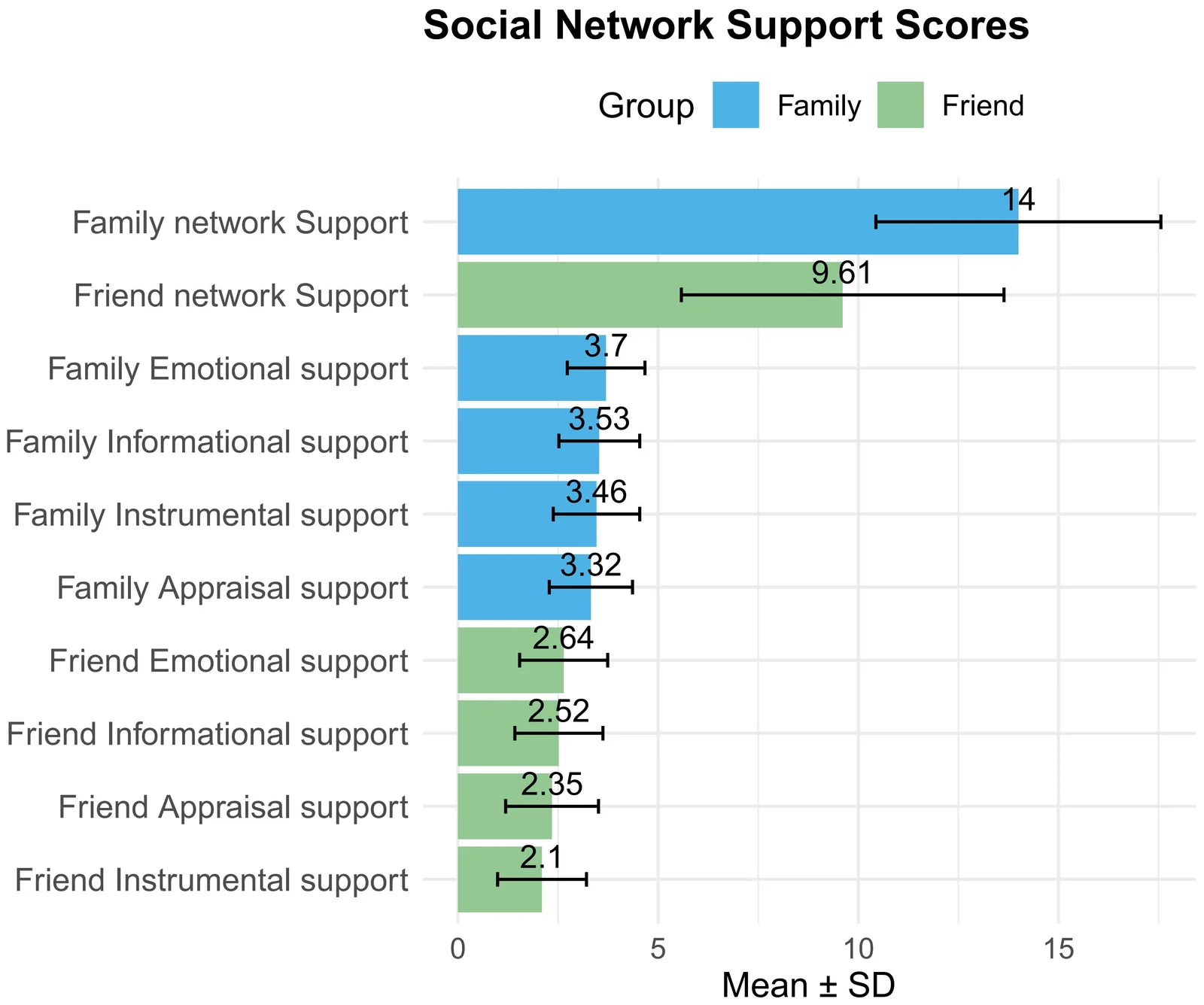
Mean ± standard deviation (SD) of overall social network support and four specific types of support (emotional, informational, instrumental, and appraisal) from family and friends among the study participants.

**Figure 2:**
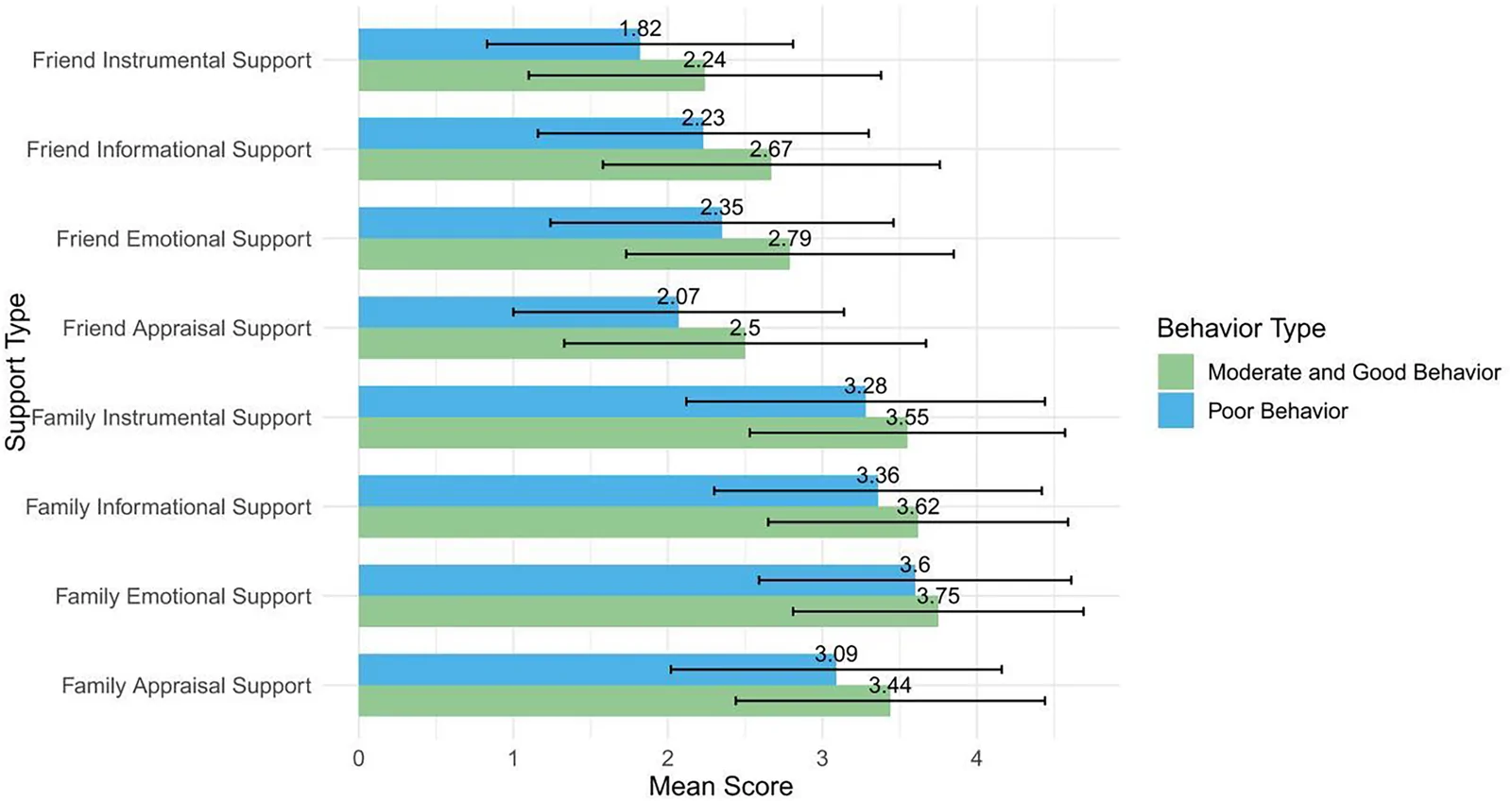
Mean ± standard deviation (SD) of family and friend social network support types among participants with poor versus moderate to good diabetes management behaviors.

### 3.2 Associations between social network properties and self-management behavior

According to the data in **Table 3**, the associations between social network characteristics and self-management behavior are evident across multiple models. After further adjustment for smoking status, alcohol consumption, and hypertension in Model 3, both family network size (*β* = 0.251, 95% *CI*: 0.048–0.455, *P* = 0.016) and friend network size (*β* = 0.325, 95% CI: 0.147–0.504, *P* < 0.001) remained significantly associated with self-management behavior. Additionally, family network support (*β* = 0.558, 95% CI: 0.406–0.710, *P* < 0.001) and friend network support (*β* = 0.624, 95% CI: 0.484–0.764, *P* < 0.001) continued to show significant positive correlations after adjustment, indicating that larger social networks and higher social support are robustly linked to better self-management behaviors. The associations between social network characteristics and self-management behavior remained consistent even after adjusting for additional confounders such as duration of diabetes, diabetes medication use, and complications. There were significant positive associations between social network characteristics and various aspects of self-management among diabetic patients. Specifically, family and friend network support were associated with improvements in diet, exercise, blood sugar testing, and foot care. Family network size was linked to better blood sugar testing, foot care, and medication monitoring, while friend network size showed associations with diet and exercise. The details of the sensitivity analyses are shown in **Supplementary Tables S2**, **S3**.

**Table 3:** Associations between social network properties and self-management behavior.

	Model 1		Model 2		Model 3	
Variables	*β (95% CI)*	*P*	*β (95% CI)*	*P*	*β (95% CI)*	*P*
Family Network size	0.307 (0.098, 0.515)	0.004	0.243(0.039, 0.447)	0.019	0.251 (0.048, 0.455)	0.016
Friend Network size	0.411 (0.233, 0.590)	<0.001	0.311(0.133, 0.489)	<0.001	0.325(0.147, 0.504)	<0.001
Family Network Support	0.551 (0.395, 0.707)	<0.001	0.561 (0.409, 0.712)	<0.001	0.558 (0.406, 0.710)	<0.001
Friend Network Support	0.784(0.646, 0.922)	<0.001	0.633 (0.494, 0.773)	<0.001	0.624 (0.484, 0.764)	<0.001

Model 1: uadjusted.

Model 2: adjusted for age, sex, marital status, ethnicity, educational level, employment status, household registration, income, and living arrangement;.

Model 3: additionally adjusted for smoking status, alcohol consumption, and hypertension.

### 3.3 Correlation analysis of social network properties, self-efficacy, and self-management behavior

As illustrated in **Figure 3**, Pearson’s correlation analysis demonstrated a significant correlation between social network characteristics, self-efficacy, and self-management behaviors (*P* < 0.001). Specifically, there was a positive correlation between social network characteristics and self-efficacy (*P* < 0.001), indicating that stronger social networks are associated with higher confidence in managing health-related tasks effectively. Moreover, social network attributes showed a positive correlation with self-management behaviors (*P* < 0.001) across the entire sample.

**Figure 3:**
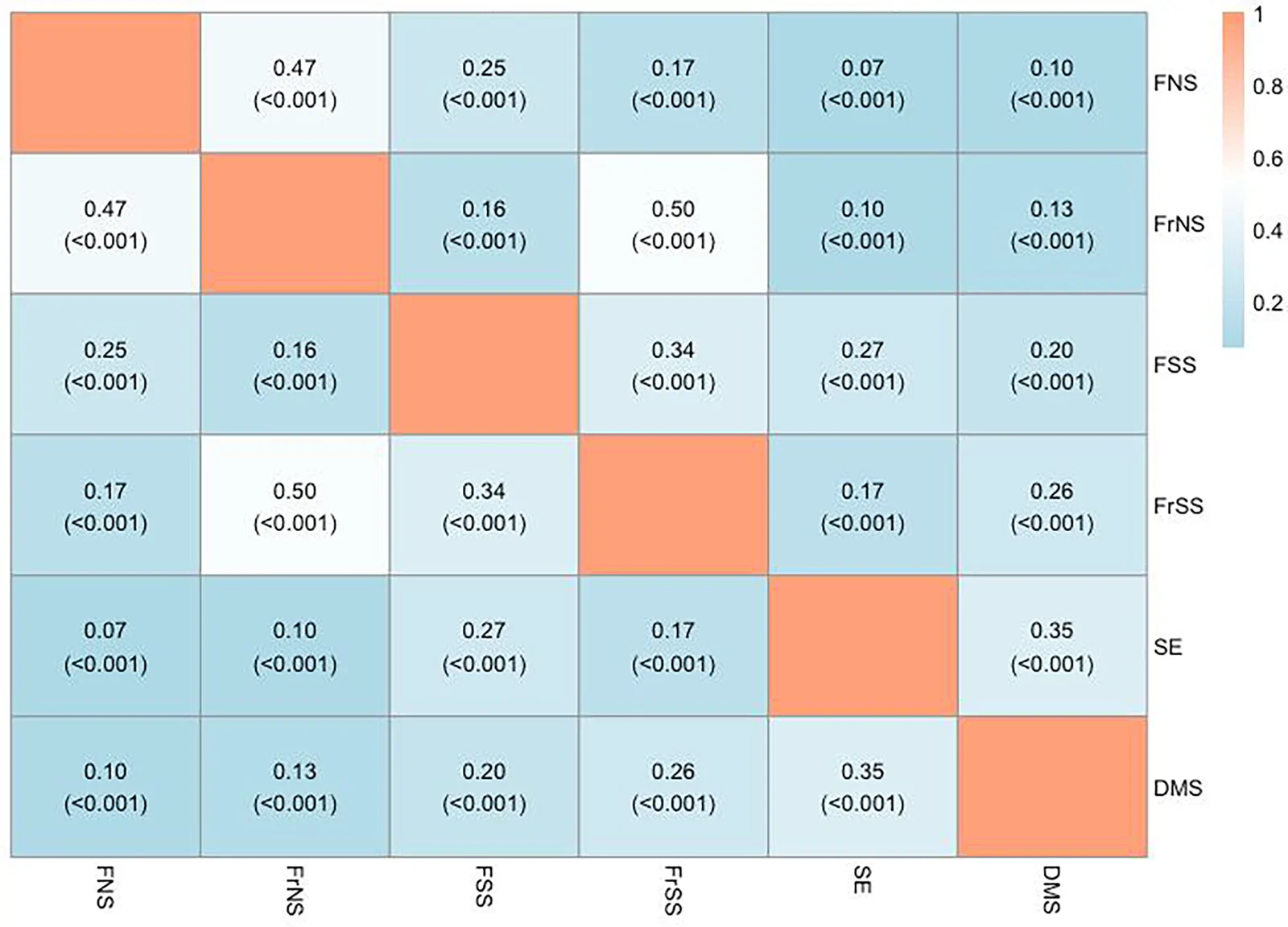
Correlation heatmap of social network properties, self-efficacy, and self-management behavior (FNS, Family Network Size; FrNS, Friend Network Size; FSS, Family Network Support; FrSS, Friend Network Support; SE, Self-Efficacy; DMS, Diabetes Management Behavior).

### 3.4 Mediating effect of self efficacy on social network properties and diabetes management behavior

Self-efficacy significantly mediates the relationship between friend network size and self-management behaviors, accounted for 28.58% of the association between friend network size and self-management behaviors.Both family and friend social support show a significant positive association with DMS, the proportion of the mediated effect is 52.32% for family social support and 13.54% for friend social support detailed results are depicted in **Figure 4**.

**Figure 4:**
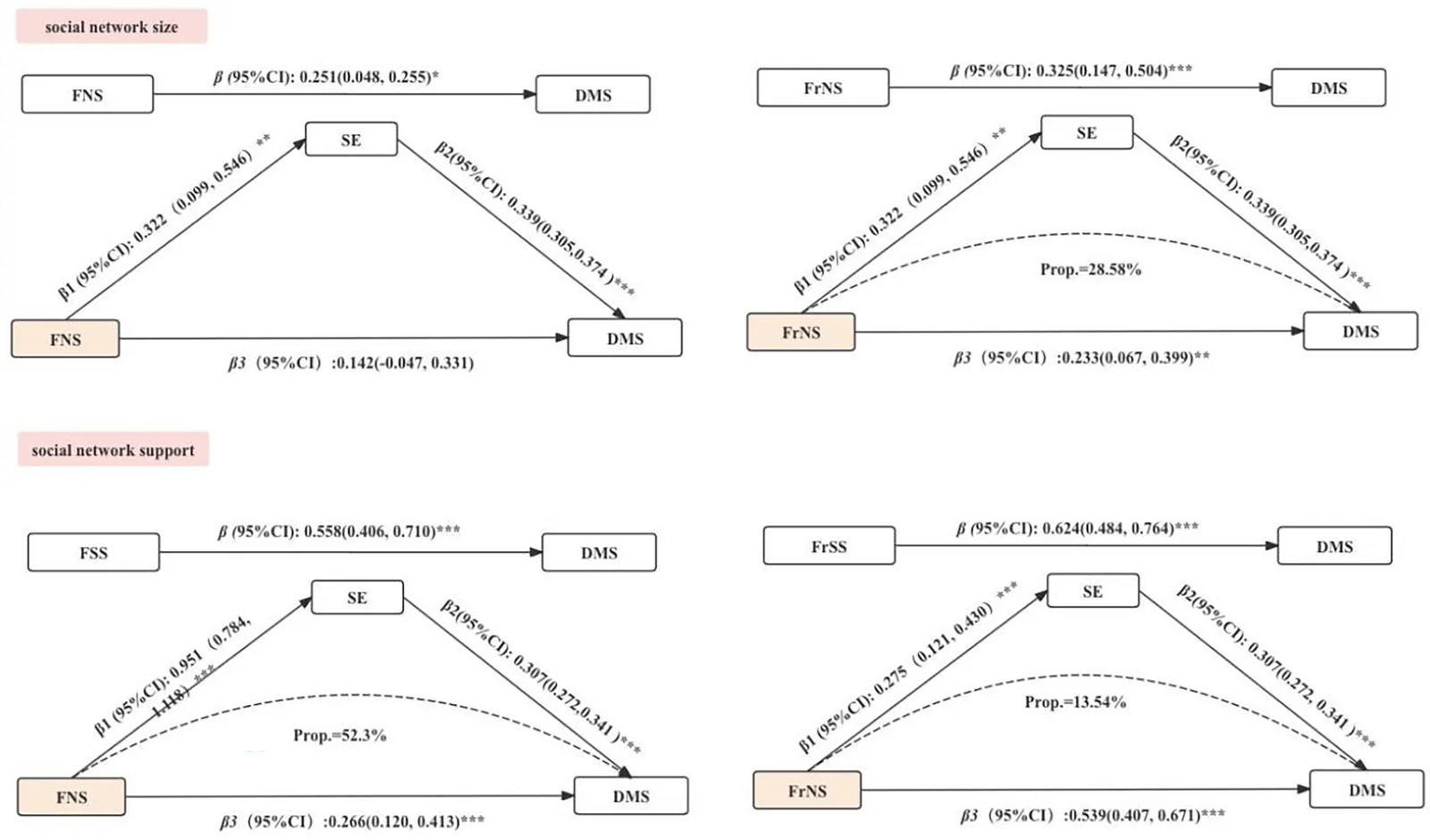
Mediating analysis between social network properties, self-efficacy, and self-management. (FNS, Family Network Size; FrNS, Friend Network Size; FSS, Family Network Support; FrSS, Friend Network Support; SE, Self-Efficacy; DMS, Diabetes Management Behavior) *<0.05, **<0.01, ***<0.001, Indirect effect; β1*β2, Direct effect; β3, Total effect.

The study conducted stratified analyses by gender and age to explore the relationship between social network characteristics and diabetes self - management behaviors, and found significant differences. In terms of gender, the friend network size of women had a significant impact on self - management behaviors, and the family network size of men had a significant impact after adjustment. Moreover, both family and friend network support had a significantly positive impact on the self - management behaviors of male and female patients. In terms of age, the friend network size, family network support, and friend network support of both people aged 60 years and above and those below 60 years were significantly and positively correlated with self - management behaviors, detailed results are depicted in **Supplementary Tables S4**, **S5**.

In addition, in different gender and age groups, self - efficacy played a significant mediating role in the relationship between social support and self - management behaviors, detailed results are depicted in **Supplementary Figures S1**–**S6**.

## 4 Discussion

The findings of this multi-center, community-based cross-sectional study highlight the intricate interplay between social network characteristics, self-efficacy, and diabetes self-management behaviors among Chinese adults. The study reveals compelling evidence that both structural (family/friend network size) and functional (support level) characteristics of social networks in diabetic patients directly influence self-management behaviors, with self-efficacy acting as a significant mediator.

The positive association between social support and self-management behaviors found in our study is consistent with global trends observed in other populations (31–33). For instance, the UK Biobank (UKB) cohort study reported a 17% higher risk of type 2 diabetes (T2DM) linked to social isolation (32), which parallels our findings of the importance of social network support. Moreover, the Mendelian randomization (MR) analysis supporting the link between frequent contact with friends or family and lower T2DM risk aligns with our results showing direct and indirect effects of family and friend support on self-management behaviors. The differential effects of family network size (β=0.251) versus friend network size (β=0.325) on self-management behaviors may relate to interaction patterns within these networks. Granovetter’s suggests that peripheral network members (e.g., friends) often provide diverse informational resources, facilitating access to alternative disease management strategies (34). This resonates with findings from David Reeves et al (35), where broad social engagement enhanced self-management through knowledge sharing. In contrast, family networks predominantly exerted influence via emotional support (e.g., encouragement and supervision), consistent with Gallant’s observation that family-driven “daily structural maintenance” critically shapes dietary and medication adherence. The stronger functional effects (family support β=0.558; friend support β=0.624) further validate the *resource mobilization hypothesis* of social capital. Vassilev et al.’s concept of *collective efficacy* (5) partially explains this phenomenon: patients’ self-management confidence increases when perceiving shared commitment from their networks. However, our findings warrant caution against over-reliance on networks, some patients may resist activating available support to preserve autonomy, implying that interventions must balance support intensity with individual agency. The gender-specific patterns observed in our study, where family support had a direct impact on both gender but friend support was more influential among males, offer novel insights. This is in contrast to some Western studies where social support from family and friends was found to be equally important for all genders (36–38). The cultural context, where males might place a higher value on peer support, could explain these differences. Our findings suggest that interventions should consider these gender nuances to be more effective.

The mediating role of self-efficacy, as identified in our study, corroborates the theoretical framework of social cognitive theory. Similar to the systematic review by Jang and Yoo (15), which emphasized the role of self-efficacy in self-management programs for chronic diseases, our results highlight the importance of enhancing self-efficacy to improve health behaviors among diabetic patients. Gallant’s systematic review (39) demonstrated that social support enhances health behaviors by fostering perceived control over disease (self-efficacy). In Chinese contexts, Yang et al (40)similarly identified self-efficacy as a mediator between social support and diabetes management. Notably, the slightly stronger mediation effect for friend support (vs. family) may reflect a “peer modeling mechanism”: friends as “similar others” provide more relatable success stories, thereby amplifying self-efficacy.

### 4.1 Limitations

While this study enriches the existing literature, it has several limitations. The cross - sectional data design restricts the ability to capture temporal changes and infer causal relationships, this cross - sectional design is unable to make causal inferences about long - term network dynamics. Although the sample is large and diverse, certain inclusion and exclusion criteria, along with potential selection biases like non - random participant recruitment, may lead to a sample that does not fully represent the broader population of diabetes patients in China. Moreover, relying on self - reported data brings the risk of reporting bias. Despite using multivariable models to adjust for known confounders, unmeasured or inadequately controlled confounders may still affect the observed relationship between social network characteristics and self - management behaviors. Additionally, there are unmeasured network heterogeneities, such as the difference between rural “family - restricted” and “diverse” network types, which may modulate mediation pathways, and the lack of social media data limits digital social capital analysis. Future studies should adopt longitudinal designs and use objective measures of self - management behaviors.

## 5 Conclusion

This study highlights the importance of social network characteristics in diabetes self-management, with a mediating role of self-efficacy. The findings call for interventions that consider the gender-specific impact of social support and aim to enhance self-efficacy to improve self-management behaviors among diabetic patients. Despite the limitations, the study provides a valuable foundation for further research and the development of targeted public health strategies.

## Data Availability

The original contributions presented in the study are included in the article/**Supplementary Material**. Further inquiries can be directed to the corresponding author.
