# Supplementary material for: Social network characteristics, self-management behavior, and the mediating role of self-efficacy among diabetes patients: a multicenter cross-sectional study
Source: Front Endocrinol (Lausanne). 2026 Sep 24;17:1934060. doi: 10.3389/fendo.2026.1934060 (PMC13647215; doi:10.3389/fendo.2026.1934060)
Supplement: Supplementary file 1 [file Supplementaryfile1.docx]

## Table SI Functional characteristics of the social network

| Variable name | Definition | Unit of measurement (possible range) |
| --- | --- | --- |
| Emotional support | Eotional support related to their diabetes condition (e.g., concern for blood glucose control, blood glucose monitoring, and medication adherence), | N (1–5) |
| Informational support | informational support regarding diabetes management (e.g., advice on diet, exercise, and medication) | N (1–5) |
| Instrumental support | Instrumental support is defined as assistance with purchasing medication, financial support, and buying glucose meters | N (1–5) |
| Appraisal support | Appraisal support is defined as encouragement and praise for improving diabetes self-management behaviors such as diet, regular medication, and exercise，feedback regarding accuracy of beliefs and appropriateness of action. | N (1–5) |
| **answered by patients:**  **How often does （family supporters) do the following? Response: Likert 1= never， 5=frequently** | | |
| How often do your family members show concern for your diabetes condition (e.g., blood glucose control, blood glucose monitoring, medication adherence)? | | |
| How often do your family members provide you with advice and guidance on diabetes management (e.g., diet, exercise, medication)? | | |
| How often do your family members assist you materially in managing your diabetes (e.g., helping with medication purchases, financial support, buying glucose meters)? | | |
| How often do your family members offer you encouragement and praise when you improve your diabetes self-management behaviors (e.g., diabetes-friendly diet, regular medication, exercise)? | | |
| **answered by patients:**  **How often does （friend supporters) do the following? Response: Likert 1= never， 5=frequently** | | |
| How often do your friends show concern for your diabetes condition (e.g., blood glucose control, blood glucose monitoring, medication adherence)? | | |
| How often do your friends provide you with advice and guidance on diabetes management (e.g., diet, exercise, medication)? | | |
| How often do your friends assist you materially in managing your diabetes (e.g., helping with medication purchases, financial support, buying glucose meters)? | | |
| How often do your friends offer you encouragement and praise when you improve your diabetes self-management behaviors (e.g., diabetes-friendly diet, regular medication, exercise)? | | |

## Table SII Between social network characteristics and various aspects of self-management behavior

| Variables | Model1 |  | Model2 |  | Model3 |  |
| --- | --- | --- | --- | --- | --- | --- |
| **Diet** | *β（*95% CI*）* | *P* | *β（*95% CI*）* | *P* | *β（*95% CI*）* | *P* |
| Family Network size | 0.105 (0.020, 0.190) | 0.016 | 0.068 (-0.017, 0.154) | 0.118 | 0.073（-0.012，0.158） | 0.091 |
| Friend Network size | 0.101 (0.028, 0.174) | 0.007 | 0.102 (0.027, 0.177) | 0.008 | 0.113（0.039，0.188） | **0.003** |
| Family Network Support | 0.133 (0.067, 0.199) | <0.001 | 0.136 (0.071, 0.201) | <0.001 | 0.139 （0.074，0.204） | **<0.001** |
| Friend Network Support | 0.206 (0.148, 0.264) | <0.001 | 0.209 (0.149, 0.268) | <0.001 | 0.196（0.136，0.255） | **<0.001** |
| **Exercise** |  |  |  |  |  |  |
| Family Network size | -0.034 (-0.096, 0.028) | 0.286 | -0.044 (-0.107, 0.019) | 0.17 | -0.04（-0.103，0.022） | 0.208 |
| Friend Network size | 0.207 (0.153, 0.260) | <0.001 | 0.188 (0.133, 0.242) | <0.001 | 0.186（0.131，0.241） | **<0.001** |
| Family Network Support | 0.077 (0.029, 0.125) | 0.002 | 0.070 (0.022, 0.118) | 0.005 | 0.070（0.022，0.118） | **0.004** |
| Friend Network Support | 0.187 (0.145, 0.230) | <0.001 | 0.156 (0.112, 0.200) | <0.001 | 0.156（0.111，0.200） | **<0.001** |
| **Blood sugar testing** |  |  |  |  |  |  |
| Family Network size | 0.065 (0.001, 0.129) | 0.045 | 0.062 (0.001, 0.122) | 0.045 | 0.063（0.002，0.123） | **0.042** |
| Friend Network size | 0.016 (-0.039, 0.071) | 0.570 | _0.039 (-0.092, 0.014) | 0.15 | -0.038（-9.091，0.015） | 0.163 |
| Family Network Support | 0.063 (0.014, 0.111) | 0.012 | 0.063 (0.017, 0.109) | 0.007 | 0.061（0.015，0.107） | **0.009** |
| Friend Network Support | 0.199 (0.155, 0.242) | <0.001 | 0.120 (0.078, 0.163) | <0.001 | 0.123（0.080，0.165） | **<0.001** |
| **Foot care** |  |  |  |  |  |  |
| Family Network size | 0.052 (-0.025, 0.129) | 0.186 | 0.077 (0.000, 0.153) | 0.049 | 0.077（0.001 ，0.153 ） | **0.048** |
| Friend Network size | 0.099 (0.033, 0.166) | 0.003 | 0.042 (-0.025, 0.109) | 0.218 | 0.046（-0.021，0.113） | 0.182 |
| Family Network Support | 0.129 (0.070, 0.187) | <0.001 | 0.146 (0.088, 0.204) | <0.001 | 0.143（0.085，0.201） | **<0.001** |
| Friend Network Support | 0.232 (0.180, 0.284) | <0.001 | 0.152 (0.099, 0.205) | <0.001 | 0.152（0.098 ，0.205） | **<0.001** |
| **Medication monitoring** |  |  |  |  |  |  |
| Family Network size | 0.118 (0.080, 0.156) | <0.001 | 0.080 (0.044, 0.116) | <0.001 | 0.078 （0.042，0.114 ） | **<0.001** |
| Friend Network size | -0.012 (-0.044, 0.021) | 0.486 | 0.018 (-0.014, 0.049) | 0.263 | 0.018 （-0.013 ，0.050） | 0.254 |
| Family Network Support | 0.150 (0.121, 0.179) | <0.001 | 0.146 (0.119, 0.173) | <0.001 | 0.145（0.117 ，0.172） | **<0.001** |
| Friend Network Support | -0.040 (-0.065, -0.014) | 0.003 | -0.003 (-0.028, 0.022) | 0.803 | -0.002（-0.027，0.023） | 0.867 |
| Model1: uadjusted。 Model2: adjusted for age, marital status, ethnicity, educational level, employment status, household registration, income, and living arrangement； Model3: additionally adjusted for smoking status, alcohol consumption, and hypertension. | | | | | | |

## **Table SIII Associations between social network properties and self-management behavior, adjusted for diabetes duration, medication use, and complications**

|  | Duration of diabetes |  | Diabetes medication use |  | Complications of diabetes |  |
| --- | --- | --- | --- | --- | --- | --- |
| Variables | *β*（*95% CI*） | *P* | *β*（*95% CI*） | *P* | *β*（*95% CI*） | *P* |
| Family Network size | 0.262 (0.059, 0.465) | **0.011** | 0.266 (0.064, 0.467) | **0.01** | 0.270 (0.069, 0.471) | **0.008** |
| Friend Network size | 0.329 (0.151, 0.507) | **<0.001** | 0.315 (0.138, 0.491) | **<0.001** | 0.292 (0.116, 0.469) | **0.001** |
| Family Network Support | 0.549 (0.397, 0.701) | **<0.001** | 0.532 (0.381, 0.683) | **<0.001** | 0.524 (0.373, 0.674) | **<0.001** |
| Friend Network Support | 0.617 (0.477, 0.757) | **<0.001** | 0.616 (0.478, 0.755) | **<0.001** | 0.619 (0.481, 0.758) | **<0.001** |
| Adjusted for age, marital status, ethnicity, educational level, employment status, household registration, income, living arrangement, smoking status, alcohol consumption, and hypertension， | | | | | | |

## **Table SIV Gender-specific associations between social network properties and self-management behavior**

|  |  | Model1 |  | Model2 |  | Model3 |  |
| --- | --- | --- | --- | --- | --- | --- | --- |
|  | Variables | *β*（*95% CI*） | *P* | *β*（*95% CI*） | *P* | *β*（*95% CI*） | *P* |
| ***female*** | Family Network size | 0.226 (-0.074, 0.527) | 0.140 | 0.204 (-0.086, 0.495) | 0.168 | 0.187 (-0.103, 0.477) | 0.206 |
|  | Friend Network size | 0.669 (0.414, 0.925) | <0.001 | 0.516 (0.262, 0.769) | <0.001 | 0.536 (0.283, 0.789) | **<0.001** |
|  | Family Network Support | 0.491 (0.265, 0.718) | <0.001 | 0.528 (0.312, 0.745) | <0.001 | 0.517 (0.300, 0.733) | **<0.001** |
|  | Friend Network Support | 0.812 (0.617, 1.006) | <0.001 | 0.604 (0.410, 0.798) | <0.001 | 0.603 (0.409, 0.796) | **<0.001** |
| ***male*** | Family Network size | 0.404 (0.115, 0.693) | 0.006 | 0.280 (-0.008, 0.568) | 0.057 | 0.304 (0.016, 0.593) | **0.038** |
|  | Friend Network size | 0.158 (-0.093, 0.409) | 0.218 | 0.113 (-0.140, 0.366) | 0.38 | 0.131 (-0.122, 0.385) | 0.309 |
|  | Family Network Support | 0.609 (0.394, 0.825) | <0.001 | 0.571 (0.357, 0.786) | <0.001 | 0.574 (0.359, 0.789) | **<0.001** |
|  | Friend Network Support | 0.754 (0.558, 0.951) | <0.001 | 0.670 (0.466, 0.873) | <0.001 | 0.655 (0.450, 0.860) | **<0.001** |
| Model1: uadjusted。 | | | | | | | |
| Model2: adjusted for age, marital status, ethnicity, educational level, employment status, household registration, income, and living arrangement； | | | | | | | |
| Model3: additionally adjusted for smoking status, alcohol consumption, and hypertension. | | | | | | | |

**Figure SI** Mediating analysis between Social Network Properties, Self-Efficacy, and Self-Management in female.（Abbreviations: FNS = Family Network Size, FrNS = Friend Network Size, FSS = Family Network Support, FrSS = Friend Network Support, SE = Self-Efficacy, DMS = Diabetes Management Behavior）*:<0.05,   **:﹤0.01,  ***:＜0.001,Indirect effect:β1*β2,Direct effect:β3,Total effect:β

**Figure SII** Mediating analysis between Social Network Properties, Self-Efficacy, and Self-Management in male.（Abbreviations: FNS = Family Network Size, FrNS = Friend Network Size, FSS = Family Network Support, FrSS = Friend Network Support, SE = Self-Efficacy, DMS = Diabetes Management Behavior）*:<0.05,   **:﹤0.01,  ***:＜0.001,Indirect effect:β1*β2,Direct effect:β3,Total effect:β

Table SV Age-stratified associations between social network properties and self-management behavior

|  |  | Model1 |  | Model2 |  | Model3 |  |
| --- | --- | --- | --- | --- | --- | --- | --- |
|  | Variables | *β（95% CI）* | *P* | *β（95% CI）* | *P* | *β（95% CI）* | *P* |
| **≥60 years** | Family Network size | 0.230 (-0.029, 0.488) | 0.082 | 0.239 (-0.014, 0.492) | 0.064 | 0.245 (-0.008, 0.497) | 0.057 |
|  | Friend Network size | 0.419 (0.192, 0.645) | <0.001 | 0.297 (0.070, 0.523) | 0.01 | 0.306 (0.080, 0.532) | **0.008** |
|  | Family Network Support | 0.566 (0.367, 0.765) | <0.001 | 0.603 (0.411, 0.796) | <0.001 | 0.604 (0.411, 0.797) | **<0.001** |
|  | Friend Network Support | 0.766 (0.595, 0.938) | <0.001 | 0.638 (0.463, 0.813) | <0.001 | 0.622 (0.447, 0.798) | **<0.001** |
| **<60 years** | Family Network size | 0.431 (0.078, 0.785) | 0.017 | 0.275 (-0.069, 0.619) | 0.117 | 0.277 (-0.068, 0.622) | 0.116 |
|  | Friend Network size | 0.404 (0.110, 0.697) | 0.007 | 0.331 (0.042, 0.621) | 0.025 | 0.355 (0.064, 0.647) | **0.017** |
|  | Family Network Support | 0.523 (0.270, 0.775) | <0.001 | 0.473 (0.227, 0.720) | <0.001 | 0.475 (0.228, 0.722) | **<0.001** |
|  | Friend Network Support | 0.823 (0.591, 1.056) | <0.001 | 0.627 (0.392, 0.861) | <0.001 | 0.644 (0.408, 0.880) | **<0.001** |
| Model1: uadjusted。 | | | | | | | |
| Model2: adjusted for age, marital status, ethnicity, educational level, employment status, household registration, income, and living arrangement； | | | | | | | |
| Model3: additionally adjusted for smoking status, alcohol consumption, and hypertension. | | | | | | | |

**Figure SIII** Mediating Analysis among Social Network Properties, Self-Efficacy, and Self-Management in People Aged 60 Years and Above.（Abbreviations: FNS = Family Network Size, FrNS = Friend Network Size, FSS = Family Network Support, FrSS = Friend Network Support, SE = Self-Efficacy, DMS = Diabetes Management Behavior）*:<0.05,   **:﹤0.01,  ***:＜0.001,Indirect effect:β1*β2,Direct effect:β3,Total effect:β

**Figure SVI** Mediating Analysis among Social Network Properties, Self-Efficacy, and Self-Management in People Under 60 Years Old。（Abbreviations: FNS = Family Network Size, FrNS = Friend Network Size, FSS = Family Network Support, FrSS = Friend Network Support, SE = Self-Efficacy, DMS = Diabetes Management Behavior）*:<0.05,   **:﹤0.01,  ***:＜0.001,Indirect effect:β1*β2,Direct effect:β3,Total effect:β

**Table SVI Investigators, Collaborators and Organization**

**Anhui Province**
Health Center of Shibali Town, Qiaocheng District, Bozhou City,

Qingxia Chen

Department of General Practice, Affiliated Suzhou Hospital of Anhui Medical University，

Weiqiang Wang

**Guangdong Province**

Chang’an Toun Community Health Service Center,Dongguan City,

Hanhai Liu, Linping Zhang

Shenzhen Nanshan District Medical Group Headquarters Tianxia Community Health Service Center,

Xianghua Li, Xiaozhou Hu

**Henan Province**

Chang'an Road Community Health Service Center, Jianxi District, Luoyang City，

Qinying Ma, Jie Jin

**Hunan** **Province**

The First People's Hospital of ChangDe City，

Cishuang Fu

**Jiangsu Province**

Xinzhen Community Health Service Center, Zhoushi Town, Kunshan City,

Luhua Xiao

**Liaoning Province**

Danan Community Health Service Center, Shenhe District, Shenyang City，

Linlin Zhang, Zhanguo Xu

**Qinghai Province**

Yunjiakou Town Health Center, Chengdong District, Xining City, Qinghai Province,

Yongxia Pu, Xiaomei Wang

Department of General Medicine, Qinghai Red Cross Hospital, No. 55, South Street, Xining City,

Xingcui Xue

Wenhui Road Community Health Service Center, Chengxi District, Xining City ,

Yinghua Xu, Xuechun Ma

**Shandong Province，**

Yangliuxue Town Heslth Cebter, Bincheng District, Binzhou City, Shandong，

Xiuhua Gong

**Shaanxi Province**

Xinhe Community Health Service Center, Baqiao District,

Baoji Wang, Jiao Jiao Fu

General Practice Department of The Second Affiliated Hospital of Xi‘an Medical University, Baqiao District，

Jia Wei, Jing Tao

**Sichuan Province**

Department of General Medicine, Chengdong Community Health Service Center, Guihu Street, Xindu District,

Longmeng Peng, Jing Tang

**Xinjiang Uygur Autonomous Region**

Department of General Medicine, Hami Central Hospital Affiliated to Xinjiang Medical University

Xiaoying Li, Dongxiang Li

Department of General Medicine, First Affiliated Hospital of Shihezi University ,Shihezi，

Lili Xu, Yinfeng Gao

Department of General Medicine, People's Hospital of Xinjiang Uygur Autonomous Region，

Abulaite Aizezi, Lulu Qin

Yunnan P**rovince**

Yunnan Third People's Hospital (Changsou Road Community Health Service Center)

Linyan Zhang, Minghui Yang

**Zhejiang Province**

Community Health Service Center of Xiasha Street,Qiantang District，

Guangxi Liu, Jingjing Huang

Department of General Practice, Quzhou People’s Hospital，

Dengpan Luo, Qipeng Wu

Department of General Practice, Shaoxing People's Hospital，

Gang Li, Zhangqing Ren

Shaoxing second hospital lizhu branch, Shaoxing，

Zhou Fang, Pingping Song

The First People's Hospital of Wuyi County

Yan Zhang

The First People's Hospital of Chun'an County、Community Health Service Center of Qiandaohu Town, Chun'an County，

Ailian Ye, Xiaobin Tang

Jianqiao Community Health Service Center, Shangcheng District，

Tao Chen, Limei Zhao
